# Loss of Hepatocyte-Specific PPAR*γ* Expression Ameliorates Early Events of Steatohepatitis in Mice Fed the Methionine and Choline-Deficient Diet

**DOI:** 10.1155/2020/9735083

**Published:** 2020-05-01

**Authors:** Jose Cordoba-Chacon

**Affiliations:** Department of Medicine, Section of Endocrinology, Diabetes and Metabolism, University of Illinois at Chicago, Chicago, IL, USA

## Abstract

The prevalence of nonalcoholic fatty liver disease (NAFLD) is increasing worldwide. To date, there is not a specific and approved treatment for NAFLD yet, and therefore, it is important to understand the molecular mechanisms that lead to the progression of NAFLD. Methionine- and choline-deficient (MCD) diets are used to reproduce some features of NAFLD in mice. MCD diets increase the expression of hepatic peroxisome proliferator-activated receptor gamma (PPAR*γ*, *Pparg*) and the fatty acid translocase (CD36, *Cd36*) which could increase hepatic fatty acid uptake and promote the progression of NAFLD in mice and humans. In this study, we assessed the contribution of hepatocyte-specific PPAR*γ* and CD36 expression to the development of early events induced by the MCD diet. Specifically, mice with adult-onset, hepatocyte-specific PPAR*γ* knockout with and without hepatocyte CD36 overexpression were fed a MCD diet for three weeks. Hepatocyte PPAR*γ* and/or CD36 expression did not contribute to the development of steatosis induced by the MCD diet. However, the expression of inflammatory and fibrogenic genes seems to be dependent on the expression of hepatocyte PPAR*γ* and CD36. The expression of PPAR*γ* and CD36 in hepatocytes may be relevant in the regulation of some features of NAFLD and steatohepatitis.

## 1. Introduction

Nonalcoholic fatty liver disease (NAFLD) is becoming the main cause of chronic liver disease, and it has a high prevalence in the general population worldwide [1]. Accumulation of fat in the hepatocytes (steatosis) associated with hepatic insulin resistance, inflammation, ballooning, and eventually fibrosis are features of NAFLD. Nonalcoholic steatohepatitis (NASH) is the advance pathological state of NAFLD, and it is characterized by hepatic inflammation and liver damage with or without fibrosis. To date, there are no FDA-approved medical treatments for NAFLD, and the prevalence of this disease is expected to keep increasing [2, 3]. Therefore, it is required to understand the metabolic processes that regulate the progression of NAFLD in order to design future treatments that reduce and reverse NAFLD. Different dietary mouse models are used to reproduce some of the features of NASH, and among them is the model of steatohepatitis induced by the methionine- and choline-deficient (MCD) diet. MCD diets induce quickly some features of NASH due in part to an increase of hepatic fatty acid uptake [4–6], reduction of hepatic fatty acid oxidation [7], secretion of very-low-density lipoprotein (VLDL) [8], and glutathione production [9, 10].

Hepatic peroxisome proliferator-activated receptor gamma (PPAR*γ*, *Pparg*) and the PPAR*γ*-regulated fatty acid translocase (FAT/CD36, *Cd36*) expression is increased in mice fed the MCD diets [5–7, 11, 12]. Both PPAR*γ* [13–15] and CD36 [16] contribute to the development of high-fat diet-induced steatosis in mice by upregulating steatogenic mechanisms that involve de novo lipogenesis (DNL) and fatty acid uptake [15, 17]. In addition, hepatic PPAR*γ* and CD36 expression is positively associated with the progression of NAFLD in mice and humans [18–20]. Previously, we have shown that adult-onset hepatocyte-specific PPAR*γ* knockout (*Pparg*^*Δ*Hep^) mice showed reduced high-fat diet-induced steatosis associated with a reduction in hepatic CD36 expression and fatty acid uptake [21]. Since, MCD diets increase the expression of hepatic PPAR*γ* and CD36 in mice, it is plausible that these genes contribute to the development of steatosis and the subsequent progression to NASH in mice fed with MCD diets. However, there are conflicts about the role that hepatic PPAR*γ* plays in the development of MCD-induced steatohepatitis since adenovirus-mediated overexpression of PPAR*γ* with a cytomegalovirus promoter (not hepatocyte-specific) reduces fibrosis and steatosis [22, 23] in mice fed an MCD diet. By contrast, hepatocyte-specific knockout of PPAR*γ* reduces high-fat diet-induced steatosis [15, 21] and proinflammatory and profibrogenic events in mouse models of alcoholic liver disease [24, 25]. Also, reduced activity of hepatic PPAR*γ*, due to inhibition of EGFR, was associated with reduced fibrosis in mice fed a fast food diet [26]. Here, we sought to assess the contribution of hepatocyte-specific PPAR*γ* and its regulated gene: CD36, in the development of MCD-induced steatohepatitis. To this end, we have used *Pparg*^*Δ*Hep^ mice with and without hepatocyte-specific CD36 overexpression. Alteration of the expression of these genes in the hepatocytes is induced in adult mice, and we assessed the early events (just 3 weeks of feeding) induced by the MCD diet in adult mice. Hepatocyte-specific PPAR*γ* and CD36 expression may not play a critical role in the development of steatosis induced by MCD diets, however, hepatocyte-specific PPAR*γ* and CD36 may contribute to the progression of steatohepatitis in adult mice.

## 2. Material and Methods

### 2.1. Mice

All mouse studies were approved by the Institutional Animal Care and Use Committee of the University of Illinois at Chicago, and they were performed in accordance with relevant guidelines and regulations of the University of Illinois at Chicago. *Pparg*^fl/fl^ mice [27] were purchased from Jackson Laboratories (Strain 004584, B3.129-Pparg^tm2Rev^/J, Bar Harbor, ME) and bred as homozygotes. *Pparg*^fl/fl^ mice were housed in a temperature (22-24°C) and humidity-controlled-specific pathogen-free barrier facility with 14 h light/10 h dark cycle (lights on at 0600 h). Mice were weaned at three weeks of age and fed a standard laboratory rodent chow diet (Formulab Diet 5008, Purina Mills, Richmond, IN), unless otherwise indicated. Ten-week-old chow-fed *Pparg*^fl/fl^ littermates were randomized and injected in the lateral tail vein with 100 *μ*l saline containing an adeno-associated vector serotype 8 (AAV8) to knock out hepatocyte PPAR*γ* expression as previously described [21]. Specifically, a group of male *Pparg*^fl/fl^ mice was injected with 1.5 × 10^11^ genome copies of AAV8 vectors that bear a thyroxine-binding globulin-driven (TBGp) Cre recombinase (AAV8-TBGp-Cre, Penn Vector Core, University of Pennsylvania), to knock out hepatocyte PPAR*γ* expression and to generate adult-onset hepatocyte-specific PPAR*γ* knockout mice (*Pparg*^*Δ*Hep^, KO). Mouse *Cd36* gene (Cat # MG50422-UT, Sino Biological Inc., Beijing, China) was cloned in an AAV8-TBGp-driven vector by Penn Vector Core to generate an AAV8-TBGp-Cd36 vector that allows the overexpression of CD36 in hepatocytes (AAV8.TBG.PI.mCd36.WPRE.bGH, Penn Vector Core). Another subset of *Pparg*^fl/fl^ mice was injected with 1.5 × 10^11^ genome copies of AAV8-TBGp-Cre and 1.5 × 10^11^ genome copies of AAV8-TBGp-Cd36 vector (*Pparg*^*Δ*Hep^+Cd36, KO+Cd36). Finally, a subset of *Pparg*^fl/fl^ mice injected with 1.5 × 10^11^ genome copies of AAV8-TBGp-Null generates controls (C).

Two weeks after AAV injections, half of the mice in each group were switched to a methionine- and choline-deficient (MCD) diet (Cat # A02082002BR, Research Diets, New Brunswick, NJ), and the other half were fed a nutrient-matched methionine- and choline-supplemented (MSD) diet (Cat # A02082003BY, Research Diets). The mice were fed MSD and MCD diets for three weeks, and then, food was withdrawn at 0700 h and mice were injected ip at 1100 h with 0.5 *μ*g 4,4-difluoro-4-bora-3a,4a-diaza-s-indacene (BODIPY)-C16 (Life Technologies)/g body weight as previously reported [28]. Blood was collected from the lateral tail vein at *t* = 0, 1, and 3 hours after BODIPY-C16 injections to determine the levels of BODIPY-C16 in plasma. Mice were killed 5 h after injection of BODIPY by decapitation, and trunk blood was collected to determine levels of NEFA, TG, cholesterol (Wako Diagnostics, Richmond VA), ALT, AST (Pointe Scientific, Canton, MI), and BODIPY-C16. The liver and fat subdepots were weighed. Livers were snap-frozen in liquid nitrogen and stored at -80°C. To measure the BODIPY-specific fluorescent signal, tissues were homogenized in radioimmunoprecipitation assay (RIPA) buffer and fluorescence was recorded (Ex 485 nm, Em 515 nm) using 10 *μ*l of plasma or a dilution of tissue supernatants in black 96-well plates.

#### 2.1.1. Assessment of Hepatic Lipids

To assess hepatic TG content, neutral hepatic lipids were extracted in isopropanol and TG measured as previously published [29]. To assess hepatic fatty acid composition, total lipids were extracted using the Bligh and Dyer method [30]. An aliquot of extracted lipids was transmethylated with BF3-methanol (Sigma-Aldrich) to quantify specific methyl esters of fatty acids using GC/MS, as we previously reported [31, 32], using 17 : 1 as the internal standard to quantify the amount of each fatty acid in the sample. In addition, we used a commercial sample of polyunsaturated fatty acid mixture (PUFA-2, Supelco) to identify the different fatty acids in the samples.

#### 2.1.2. Gene Expression Analysis

Hepatic RNA was extracted using the TRIzol Reagent (Life Technologies, Carlsbad, CA) and treated with RQ1 RNase-free DNase (Promega, Madison, WI). DNA-free RNA was transcribed, and qPCR was performed as previously published [29, 31]. Peptidylprolyl isomerase (*Ppia*), *β*-actin (*Actb*), and hypoxanthine-guanine phosphoribosyltransferase (*Hprt*) were used as housekeeping genes to calculate a normalization factor, as previously reported [29]. qPCR primer sequences of *Ppia*, *Actb*, *Hprt*, *Pparg*, *Cd36*, tumor necrosis factor alpha (*Tnfa*), transforming growth factor beta 1 (*Tgfb1*), alpha smooth muscle actin (*aSma*), and collagen 1a1 (*Col1a1*) were published previously [28]. Primer sequences of F4/80 (NM_010130.4) Se: AGTACGATGTGGGGCTTTTG, As: TCTGTGGTGTCAGTGCAGGT, 164 bp; metalloproteinase 13 (*Mmp13*, NM_008607.2) Se: ATCCCTTGATGCCATTACCA, As: GCCCAGAATTTTCTCCCTCT, 204 bp; and TIMP metallopeptidase inhibitor 1 (*Timp1*, NM_001294280.2) Se: CCAGAACCGCAGTGAAGAG, As: CTCCAGTTTGCAAGGGATAGA, 193 bp.

#### 2.1.3. Hematoxylin and Eosin and Picrosirius-Red Fast-Green Staining

Livers were fixed in formalin (Fisher Scientific) for 48 h. Fixed livers were paraffin embedded, and 5 *μ*m unstained and hematoxylin and eosin-stained liver sections were prepared by the Research Histology and Tissue Imaging Core of the University of Illinois at Chicago. In order to stain collagen fibers, liver sections were deparaffinized, hydrated in graded-ethanol/water solutions, and then stained in a solution of 0.1% Direct Red (Cat # 365548, dye content > 25%, Sigma-Aldrich) and 0.1% Fast Green FCF (Cat # P6744, dye content > 85%, Sigma-Aldrich) in saturated picric acid for 60 minutes, followed by 0.5% acetic acid solution for 5 minutes. Samples were quickly dehydrated and mounted with Permount Mounted Media (Fisher Chemical). Pictures were taken with an inverted Microscope DMi8 and the Leica Application Suite X software (Leica microsystems CMS GmbH). The Sirius red-stained area was quantified with ImageJ (NIH, Bethesda, MD).

#### 2.1.4. Statistics

Values are represented as means ± standard errors of the mean (SEM). Two-way ANOVA followed by Tukey's post-test was used. Due to variability of hepatic CD36, expression was log-transformed for statistical analysis. The statistical analysis was performed using GraphPad Prism 8 (GraphPad Software, La Jolla, CA). *p* values less than 0.05 were considered significant.

## 3. Results

### 3.1. Expression Levels of Hepatic PPAR*γ* and CD36 Did Not Alter Body Composition or Plasma Lipids in Mice Fed a MCD Diet

**Figure 1:**
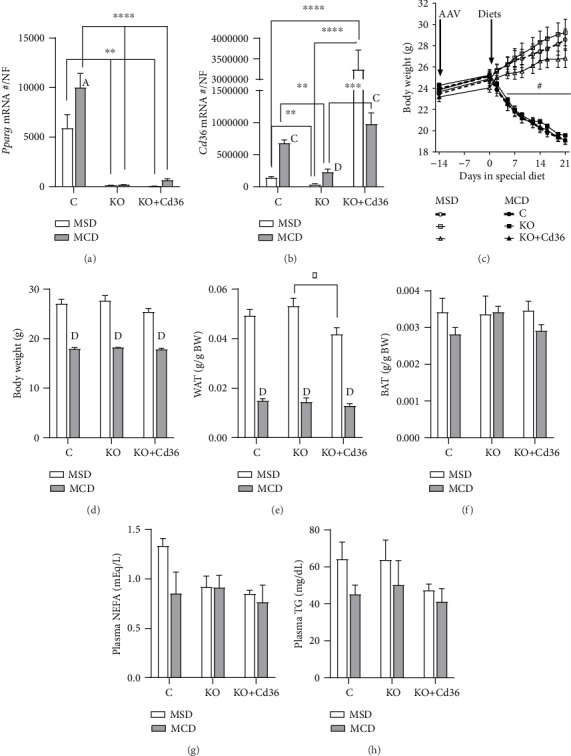
Effect of MCD diet on body composition, plasma lipids, and ALT levels of *Pparg*^*Δ*Hep^ mice and *Pparg*^*Δ*Hep^ mice with overexpression of hepatocyte CD36. Hepatic expression of (a) *Pparg* and (b) *Cd36*. Gene expression is represented as an absolute copy number normalized by the normalization factor (NF). (c) Changes in body weight induced by MSD and MCD diets. (d) Body weight at sac. (e) Relative white adipose tissue (WAT) weight. The weight of WAT is the sum of urogenital and subcutaneous adipose tissue weights. (f) Relative brown adipose tissue (BAT) weight. Plasma (g) NEFA and (h) TG levels. Values are represented as the mean ± standard error of the mean. Letters or ^#^ represents significant differences between MSD and MCD within the group. Asterisks indicate significant differences between groups within the same diet. ^∗^,^A^,^#^*p* < 0.05; ^∗∗^,^B^*p* < 0.01; ^∗∗∗^,^C^*p* < 0.001; ^∗∗∗∗^,^D^*p* < 0.0001. Control mice (C, circles); *Pparg*^*Δ*Hep^ mice (KO, squares); *Pparg*^*Δ*Hep^ mice with hepatocyte CD36 overexpression (KO+Cd36, triangles). MSD diet: open columns, open symbols; MCD diet: close columns, close symbols. *n* = 3‐7 mice/group.

In order to assess the role of hepatocyte PPAR*γ* in the early events of steatohepatitis induced by MCD diet, we have knocked out the expression of PPAR*γ* only in hepatocytes of adult mice and fed the mice with MCD diet for only three weeks. MCD diet increased the expression of hepatic PPAR*γ* and CD36 in PPAR*γ*-intact mice (Figures 1(a) and 1(b)), and the expression of hepatic PPAR*γ* and CD36 was dramatically reduced in *Pparg*^*Δ*Hep^ mice (Figures 1(a) and 1(b)). We published previously that the expression of hepatic PPAR*γ* mRNA and protein was reduced with a single injection of AAV8-TBGp-Cre [21]. To assess the contribution of hepatocyte CD36 independently of that of hepatocyte PPAR*γ* to the development of early events of steatohepatitis in mice fed a MCD diet, we overexpressed physiological levels of hepatocyte CD36 in *Pparg*^*Δ*Hep^ mice (KO+Cd36), as shown by the levels of CD36 mRNA and protein (Figure 1(b), Supplementary Materials). As expected, the mice that were fed the MCD diet showed a dramatic reduction in body weight [7, 8] that was independent of the expression of hepatocyte PPAR*γ* and CD36 (Figures 1(c) and 1(d)). We just fed MCD diets only for three weeks to assess the early changes in body composition and steatohepatitis. The reduction in body weight was associated with a dramatic reduction in relative white adipose tissue but not brown adipose tissue (Figures 1(e) and 1(f)). Interestingly, the MCD diet did not alter the levels of plasma NEFA or TG levels (Figures 1(g) and 1(h)). In sum, we altered the expression of PPAR*γ* and CD36 in hepatocytes of adult *Pparg*^fl/fl^ mice, but that did not alter the effect of MCD diets on adiposity or plasma lipids. However, it may be possible that the role of hepatocyte PPAR*γ* and CD36 in MCD-fed mice is restricted to specific processes of hepatic lipid metabolism and/or the progression of steatohepatitis.

### 3.2. Hepatocyte PPAR*γ* and CD36 Are Dispensable for the Development of Steatosis in MCD-Fed Mice

**Figure 2:**
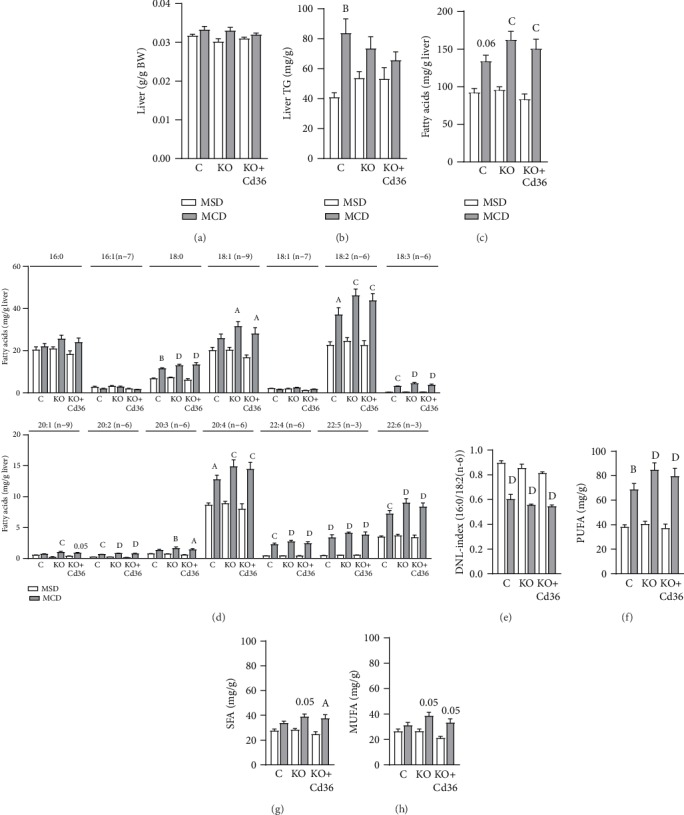
MCD diet increased hepatic polyunsaturated fatty acids independently of hepatocyte PPAR*γ* and CD36 expression. (a) Relative liver weight. (b) Hepatic triglycerides (Liv TG) levels. Hepatic levels of (c) total fatty acid levels, (d) individual subspecies of fatty acid methyl esters, (e) de novo lipogenesis (DNL: 16/18 : 2 (*n*‐6)) index, (f) saturated fatty acids (SFA), (g) monounsaturated fatty acids (MUFA), and (h) polyunsaturated fatty acids (PUFA). Values are represented as the mean ± standard error of the mean. Letters represent significant differences between MSD and MCD within the group. ^A^*p* < 0.05; ^B^*p* < 0.01; ^C^*p* < 0.001; ^D^*p* < 0.0001. Control mice (C); *Pparg*^*Δ*Hep^ mice (KO); *Pparg*^*Δ*Hep^ mice with hepatocyte Cd36 overexpression (KO+Cd36). MSD diet: open columns; MCD diet: close columns. *n* = 5‐6 mice/group.

**Figure 3:**
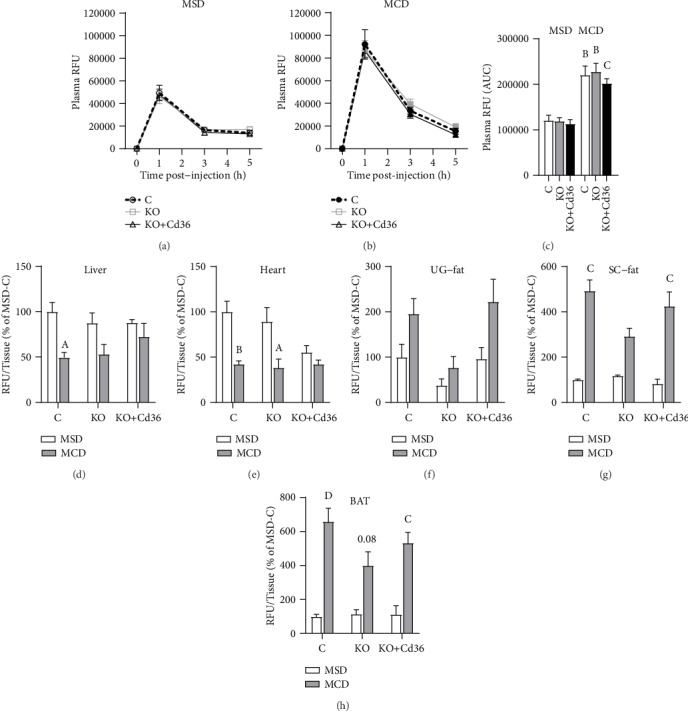
MCD diet reduced hepatic fatty acid uptake but increased fatty acid uptake in adipose tissue. Plasma BODIPY-C16 levels in mice fed (a) MSD diet or (b) MCD diet. (c) Area under the curve of plasma BODIPY-C16 levels. (d) Liver-, (e) heart-, (f) urogenital (UG) fat-, (g) subcutaneous (SC) fat-, and (h) brown adipose tissue- (BAT-) specific uptake of BODIPY-C16. RFU: relative fluorescence units. Values of RFU/tissue are represented as the percentage of control mice fed a MSD diet (e–h). Letters represent significant differences between MSD and MCD within groups. ^A^*p* < 0.05; ^B^*p* < 0.01; ^C^*p* < 0.001; ^D^*p* < 0.0001. Control mice (C); *Pparg*^*Δ*Hep^ mice (KO); *Pparg*^*Δ*Hep^ mice with hepatocyte Cd36 overexpression (KO+Cd36). MSD diet: open columns, open symbols; MCD diet: close columns, close symbols. In (a–c), control mice (open columns, discontinuous lines), KO mice (grey lines and columns), and KO+Cd36 mice (black lines and columns). *n* = 4‐7 mice/group.

Hepatocyte PPAR*γ* and CD36 play a significant role in the storage of lipids in the liver [16, 21]. It has been proposed that hepatic PPAR*γ* and CD36 may increase the uptake of lipids by hepatocytes which could promote steatosis in mice fed a MCD diet. To the best of our knowledge, this is the first study that assessed the role of hepatocyte PPAR*γ* and CD36 expression in steatosis of adult mice fed a MCD diet with the use of *Pparg*^fl/fl^ mice. Although 3 weeks of MCD diet did not increase significantly liver weight in this study, there was a positive effect of MCD diet on relative liver weight (*p* = 0.0098), which was associated with an increase in hepatic triglycerides (Figures 2(a) and 2(b), Liv TG, MCD-effect, *p* = 0.0004). Of note, the increase of hepatic TG was significant only in PPAR*γ*-intact mice. However, when we measured the composition of hepatic fatty acids by GC/MS which include those in neutral (TG) and polar lipids (mainly phospholipids), the total amount of fatty acids was increased in MCD-fed mice independent of hepatic PPAR*γ* and CD36 expression (Figure 2(c)). The fatty acids that can be generated *in situ* by hepatic DNL: palmitic acid (16 : 0), palmitoleic acid (16 : 1 n-7), and oleic acid (18 : 1 n-7), were not increased in MCD-fed mice (Figure 2(d)). As suggested by other studies, the MCD diet may reduce hepatic DNL and the levels of hepatic saturated (SFA) and monounsaturated (MUFA) fatty acids. In this study, we assessed the rate of hepatic DNL indirectly, by measuring the ratio of specific fatty acids which are known to be indicative of the level of DNL in the liver [33, 34], and found that the hepatic DNL index (ratio of 16 : 0 and 18 : 2 (*n*‐6)) was significantly reduced in MCD-fed mice (Figure 2(e)). By contrast, the absolute levels of hepatic polyunsaturated fatty acids (PUFA), which cannot be synthetized by DNL, were dramatically increased in MCD-fed mice, whereas SFA and MUFA were slightly increased by MCD diet in *Pparg*^*Δ*Hep^ mice with or without CD36 overexpression (Figures 2(f)–2(h)). The selective accumulation of PUFA in mice that were fed the MCD diet may be the consequence of increased uptake of fatty acids and/or reduced release of VLDL by the liver as published by others [8]. To assess if tissue-specific fatty acid uptake was altered by the MCD diet, we measured the uptake of fatty acids in different tissues using BODIPY-C16 (fluorescence-labeled palmitate) as an indicator of fatty acid uptake. Interestingly, the clearance of plasma BODIPY-C16 in mice fed with the MCD diet was impaired (Figures 3(a)–3(c)), and this was associated with reduced uptake of fatty acids by the liver and heart (Figures 3(d) and 3(e)). Conversely, subcutaneous white adipose tissue and brown adipose tissue showed increased uptake of fatty acids (Figures 3(f)–3(h)). Although the uptake of fatty acids by the adipose tissue was increased, that may not be enough to compensate for the reduced uptake of fatty acids in other tissues resulting in a delayed clearance of exogenous-labeled fatty acids. Also, the impaired clearance of BODIPY-C16 could be a consequence of the dramatic reduction in the amount of adipose tissue in mice that were fed the MCD diet as compared to that of mice fed the MSD (with intact adipose tissue). Overall, these data suggested that MCD diets promoted steatosis and altered the composition of fatty acids in the liver independently of hepatocyte PPAR*γ* or CD36 expression and hepatic fatty acid uptake. However, PPAR*γ* and/or CD36 expression may be involved in the progression of steatohepatitis in MCD-fed mice by the promotion of inflammation and fibrosis.

### 3.3. Expression of Hepatocyte CD36 in *Pparg*^*Δ*Hep^ Is Sufficient to Promote Inflammatory and Fibrogenic Gene Expression in Livers of MCD-Fed Mice

**Figure 4:**
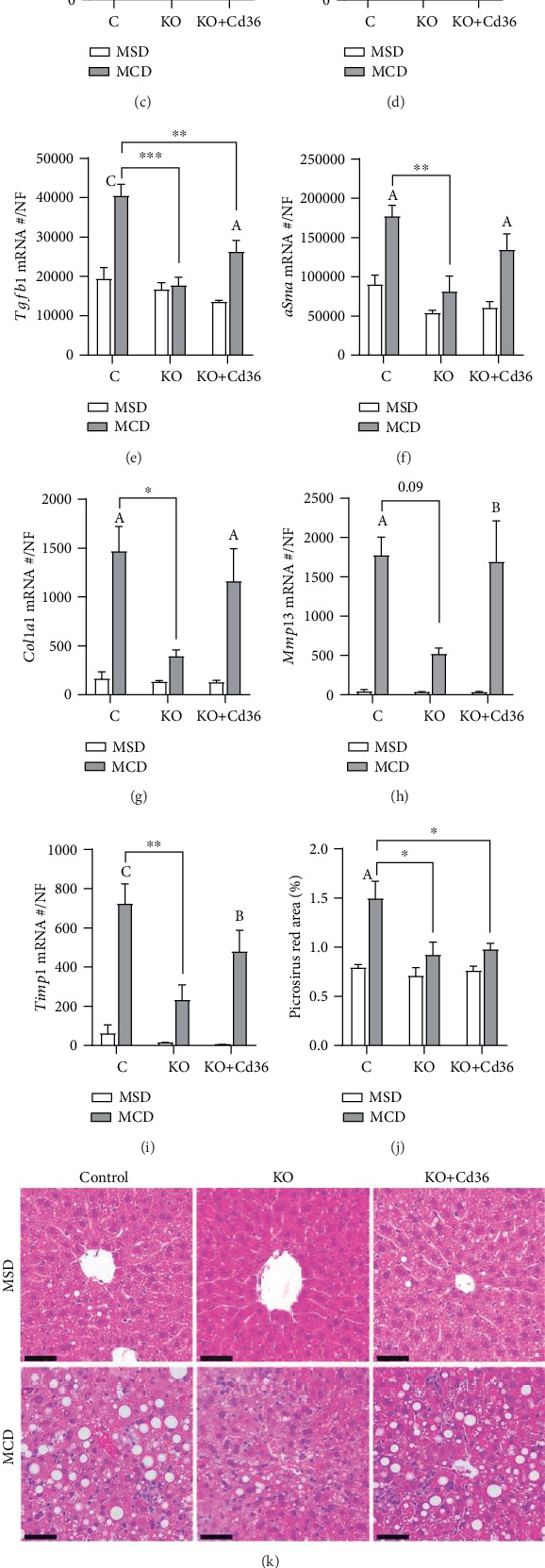
*Pparg* ^*Δ*Hep^ reduced MCD-induced fibrogenesis. Plasma (a) ALT and (b) AST levels. Hepatic expression of (c) tumor necrosis factor alpha (*Tnfa*), (d) *F4/80*, (e) transforming growth factor beta 1 (*Tgfb1*), (f) alpha smooth muscle actin (*aSma*), (g) collagen 1a1 (*Col1a1*), (h), metalloproteinase 13 (*Mmp13*), and (i) TIMP metallopeptidase inhibitor 1 (*Timp1*). (j) Quantification of picrosirius red area represented as percentage of red-stained area. (k) 20x representative images of hematoxylin and eosin-stained liver sections. (l) 10x representative images of picrosirius red/fast green-stained liver sections. Values are represented as the mean ± standard error of the mean. Hepatic gene expression is represented as an absolute mRNA copy number normalized by a normalization factor (NF). Letters represent significant differences between MSD and MCD within groups. Asterisks indicate significant differences between groups within the same diet. ^∗^,^A^*p* < 0.05; ^∗∗^,^B^*p* < 0.01; ^∗∗∗^,^C^*p* < 0.001. Control mice (C); *Pparg*^*Δ*Hep^ mice (KO); *Pparg*^*Δ*Hep^ mice with hepatocyte Cd36 overexpression (KO+Cd36). MSD diet: open columns, open symbols; MCD diet: close columns, close symbols. *n* = 3‐7 mice/group. Bar = 50 *μ*M (k); 100 *μ*M (l).

It is well-known that MCD diets promote steatohepatitis in mice. Plasma alanine aminotransferase (ALT, Figure 4(a)) was increased, while plasma aspartate aminotransferase (AST, Figure 4(b)) was not significantly increased in MCD-fed mice. As shown by others, the induction of plasma ALT occurs in the first weeks of MCD feeding and plasma AST rises progressively with longer MCD feeding [35]. To determine if the increase in plasma ALT levels was leading to the upregulation of proinflammatory and profibrogenic genes, we measured the expression of hepatic tumor necrosis factor alpha (*Tnfa*), *F4/80*, transforming growth factor beta 1 (*Tgfb1*), alpha smooth muscle actin (*aSma*), collagen 1a1 (*Col1a1*), metalloproteinase 13 (*Mmp13*), and TIMP metallopeptidase inhibitor 1 (*Timp1*). Control mice that were fed the MCD diet showed a significant increase in the expression of these genes related to the development of inflammation and fibrosis in NASH. However, *Pparg*^*Δ*Hep^ mice fed the MCD diet showed a significant reduction in the expression of *Tnfα*, *Tgfβ1*, *αSma*, *Col1a1*, *Mmp13*, and *Timp1* as compared with that of MCD-fed controls (Figures 4(c)–4(i)). Surprisingly, the overexpression of hepatocyte CD36 in *Pparg*^*Δ*Hep^ mice was associated with an increase in the expression of *Tnfα*, *F4/80*, *αSma*, *Col1a1*, *Mmp13*, and *Timp1* to levels similar to those observed in MCD-fed controls (Figures 4(c)–4(i)). Overall, these results suggest that the expression of hepatocyte-specific PPAR*γ*, and interestingly the expression of CD36, may promote the inflammatory and fibrogenic response to MCD diet by nonparenquimal cells: immune cells and hepatostellate cells. These early changes in the expression of profibrogenic hepatic genes of mice fed the MCD diet for three weeks were confirmed with the quantification of collagen in picrosirius red/fast green-stained liver sections (Figure 4(j)). In addition, the hematoxylin and eosin- and picrosirius red and fast green-stained liver sections indicate that the MCD diet induced a reorganization of the hepatic histology that includes macrovesicular steatosis, mild inflammation, and fibrosis (Figures 4(k) and 4(l)), which supported the data obtained from hepatic gene expression.

Taken together, although *Pparg*^*Δ*Hep^ did not reduce the levels of plasma ALT nor steatosis, these data indicated that hepatocyte PPAR*γ* and CD36 expressions could contribute to the upregulation of genes related to the progression of NASH. Since proinflammatory and profibrogenic genes are expressed in nonparenchymal cells, these data also suggested that some type of communication between parenchymal and nonparenchymal cells may be altered by PPAR*γ* and CD36 that facilitates the development of early events of steatohepatitis in mice fed the MCD diet.

## 4. Discussion

Hepatic PPAR*γ* expression is positively associated with the development of NAFLD in mice and humans [19, 36, 37]. Specifically, it has been proposed that the upregulation of PPAR*γ* and CD36 in NAFLD might increase hepatic lipid uptake and promote the development of steatosis [3, 17]. MCD-fed mice are a classic model of diet-induced steatohepatitis [9, 10], and the expression of hepatic PPAR*γ* and CD36 is increased in mice fed with a MCD diet [5–7, 11, 12]. Therefore, based on their known actions on lipid metabolism and homeostasis in the liver, PPAR*γ* and CD36 may increase lipid uptake in hepatocytes and contribute to the progression of steatosis and steatohepatitis induced by MCD diets. In this study, we have taken advantage of the use of our inducible hepatocyte-specific PPAR*γ* KO (*Pparg*^*Δ*Hep^) mouse model to test the relevance of hepatocyte PPAR*γ* in the development of MCD-induced steatohepatitis in adult mice. Also, we have overexpressed CD36 in hepatocytes of *Pparg*^*Δ*Hep^ mice to study the effects of CD36 in the progression of the disease independently of PPAR*γ* expression.

PPAR*γ* regulates steatogenic mechanisms that lead to fat deposition in hepatocytes in mice [38, 39]. Hepatocyte-specific PPAR*γ* knockout mice have shown that PPAR*γ* is required to increase the expression of acetyl-CoA carboxylase (*Acc1*), fatty acid synthetase (*Fasn*), stearoyl-CoA desaturase 1 (*Scd1*), *Cd36*, monoacylglycerol O-acyltransferase (Mogat1), and fatty acid-binding protein 1 (*Fabp1*) [14, 15, 21], which are genes involved in DNL and lipid uptake. However, the development of MCD-induced steatosis is not dependent on DNL, since the MCD diet reduces the levels of insulin, glucose, expression of hepatic DNL enzymes, and hepatic DNL rate [8, 40]. Also, mice fed the MCD diet showed reduced amounts of hepatic SFA and MUFA which are produced mainly by DNL and increased hepatic levels of PUFA [4, 40], which are not synthetized by DNL but modified from preformed PUFA absorbed from the diet. Our results are in line with previous reports that suggest that hepatic steatosis is independent of DNL in mice fed the MCD diet. However, the enrichment of hepatic PUFA in MCD-fed mice might be the consequence of increased hepatic fatty acid uptake [8]. Hepatocyte-specific PPAR*γ* contributes to increase hepatic lipid uptake likely by upregulating CD36 expression. PPAR*γ* binds to the promoter of CD36 and increases its expression [41, 42], which is associated with the development of hepatic steatosis. In fact, the adenovirus-mediated overexpression of hepatic CD36 led to steatosis in chow-fed mice [43], and hepatocyte-specific knockout of CD36 reduced hepatic lipid uptake and steatosis in a model with diet-induced steatosis [16], which support the steatogenic role of hepatocyte CD36. Hepatic CD36 expression is increased in hepatocytes of mice fed the MCD diet [6, 7], and it has been proposed that livers of mice fed a MCD diet take the excess fatty acids released by the white adipose tissue, and that leads to the development of steatosis [4–6, 8]. However, the dramatic loss of adipose tissue induced by MCD diets may reduce the net flux of fatty acids from adipose tissue to the liver over time [7], and as a consequence, the potential contribution of hepatocyte CD36 to the development of MCD-induced steatosis. Also, it has been shown that methionine deprivation increases energy expenditure and reduced resting respiratory quotient [40, 44], suggesting an increased utilization of lipids as a source of energy in peripheral tissues. In fact, a “browning” effect of the MCD diet on white adipose tissue associated with the upregulation of uncoupled protein 1 has been described previously [4, 12]. Overall, the net contribution of white adipose tissue lipolysis to steatosis in mice fed the MCD diet might be reduced over time due to increased oxidation in peripheral tissues and limited net availability of NEFA to the liver. Our data would be in line with these observations and would support that the MCD diet increases fatty acid uptake and utilization in adipose tissue which would reduce the net flux of fatty acids to the liver. Therefore, the sustained increased expression of CD36 in the liver may not be required for the progression of steatosis in mice with steatohepatitis [7, 8].

Steatosis is the major hallmark of NAFLD, but the progression of steatosis to NASH requires the development of inflammation that may be associated with fibrosis. The role of hepatocyte-specific PPAR*γ* in inflammation and fibrogenesis is poorly understood. This is in part due to the attributed low expression of PPAR*γ* in hepatocytes and the well-known anti-inflammatory and antifibrotic effects of PPAR*γ* in nonparenchymal cells: macrophages and in hepatic stellate cells [45]. A previous study has shown that overexpression of PPAR*γ* using a cytomegalovirus promoter (not hepatocyte-specific) in mice fed MCD diet reduces fibrosis [22, 23]. This effect may be due to the expression in nonparenchymal hepatic cells that includes hepatic stellate cells where PPAR*γ* serves as an antifibrogenic factor, and macrophages where PPAR*γ* serves as an anti-inflammatory factor. These protective actions of hepatic PPAR*γ* were previously described in a model of liver injury induced by CCl4 [45]. In addition, cytomegalovirus promoter-mediated expression of PPAR*γ* in white adipose tissue due to extrahepatic infection of adenovirus particles could increase the insulin-sensitizing effects of PPAR*γ* and reduce indirectly hepatic lipid accumulation [46]. However, in striking contrast, in a model of high-fat diet plus binge ethanol, hepatocyte-specific PPAR*γ* KO reduced the expression of collagens and the staining of collagen fibers [24]. In addition, EGFR inhibitor-mediated reduction of hepatic PPAR*γ* activity (mainly in hepatocytes) was associated with reduced and reversed steatosis and fibrosis in a mouse model of NASH induced with fast food diet [26]. Therefore, our data would add to previous observations that suggest a potential pathological role of hepatocyte-specific PPAR*γ* expression in the development of steatohepatitis.

In our study, we have knocked out specifically the expression of PPAR*γ* in hepatocytes of adult mice by using a Cre recombinase driven by a hepatocyte-specific promoter, and *Pparg*^*Δ*Hep^ mice showed reduced induction of fibrogenesis in the early stages of steatohepatitis induced by the MCD diet. Furthermore, our study suggested that hepatocyte PPAR*γ* contribution to the progression of NASH may be independent of steatosis. These results have translational relevance since the expression of PPAR*γ* in humans is associated with the progression of NASH [19, 36, 37] and the expression of the PPAR*γ*-regulated CD36 is increased in humans with NASH [18]. To date, the pharmacological activation of PPAR*γ* with Thiazolidinediones (TZDs) and the use of novel TZDs with reduced ability to bind PPAR*γ* have been studied as a potential therapy to reverse NASH and steatosis [47–51]. However, although modest therapeutic effects of TZD on steatosis of patients with NASH have been consistently reported, there is not a consensus in the effects that pharmacological activation of PPAR*γ* may have on fibrosis in patients with NASH. Therefore, it is possible that the anti-NASH effects of TZD, which are based mainly on their insulin-sensitizing effects, may be offset in somehow by the activation of hepatocyte-specific PPAR*γ* by endogenous ligands and/or TZD. This study suggests that specific expression of PPAR*γ* in hepatocytes of mice fed the MCD diet may facilitate proinflammatory and profibrogenic mechanisms, in part via expression of CD36, that in somehow promote NASH. However, further investigations are required to elucidate the mechanisms regulated by hepatocyte-specific PPAR*γ* and if they play a role in the interplay between hepatocytes and nonparenchymal cells, that may offset the therapeutic effects of whole-body PPAR*γ* activation in patients with NASH.

In sum, we have assessed the contribution of hepatocyte-specific PPAR*γ* and CD36 expression in the early events of steatohepatitis induced by the MCD diet. Despite steatosis observed in MCD-fed mice is thought to be promoted by enhanced lipid uptake, in part, due to increased hepatocyte PPAR*γ* and CD36 expression, our data suggested that PPAR*γ* and/or CD36*-*dependent lipid uptake is not a major mechanism required for the development of steatosis in a model of steatohepatitis induced by the MCD diet. However, the expression of these genes in hepatocytes may be required to promote fibrosis in mice fed the MCD diet.

## Data Availability

The data used to support the findings of this study are available from the corresponding author upon request.

## Supplementary Materials

Supplementary Materials Expression of hepatic CD36 protein in the control (C), *Pparg*^*Δ*Hep^ (KO), and *Pparg*^*Δ*Hep^ with overexpression of hepatocyte CD36 (KO+Cd36) mice.
