# Supplementary material for: Loss of Hepatocyte-Specific PPARγ Expression Ameliorates Early Events of Steatohepatitis in Mice Fed the Methionine and Choline-Deficient Diet
Source: PPAR Res. 2020 May 1;2020:9735083. doi: 10.1155/2020/9735083 (PMC7211257; doi:10.1155/2020/9735083)
Supplement: Supplementary Materials — Expression of hepatic CD36 protein in the control (C), PpargΔHep (KO), and PpargΔHep with overexpression of hepatocyte CD36 (KO+Cd36) mice. [file 9735083.f1.docx]

**Loss of hepatocyte-specific PPARγ expression ameliorates early events of steatohepatitis in mice fed the methionine and choline deficient diet.**

Jose Cordoba-Chacon, PhD

Department of Medicine, Section of Endocrinology, Diabetes, and Metabolism, University of Illinois at Chicago, Chicago, IL.

**Expression of hepatic CD36 protein in Control (C), *Pparg*^ΔHep^ (KO), and *Pparg*^ΔHep^ with overexpression of hepatic CD36 (KO+Cd36) mice.**

As described below in “*Protein extraction and Western blot”,* proteins were extracted from frozen livers and western blots were performed to detect the amount of hepatic CD36 in these blots (see representative lane of blots on the left panel), and β–actin as housekeeping protein. Samples of MSD-fed mice and those of MCD-fed mice were run in different blots, and the values were not compared between them due o difference in transfer efficiency. The overexpression of hepatic CD36 in *Pparg*^ΔHep^ mice (KO+Cd36) was significant increased in mice fed the MSD and the MCD diet as compared to that of *Pparg*^ΔHep^ mice (KO, right panel). Data are represented as mean +/- SEM. Values were compared by a one-way ANOVA followed by a Tukey post-test. *, p<0.05. n=4-5 mice/group.

**Protein extraction and Western blot:** Livers were homogenized in extraction buffer pH 7.5, 50 mM HEPES, 2 mM EGTA, 2 mM EDTA, 130 mM NaCl, 10 mM NaF, 20 mM β-glycerophosphate, 2 mM sodium pyrophosphate, 1 mM sodium vanadate, 0.5 mM PMSF, 0.1% nonidet P-40, 2 mM benzamidine, 1 mM TLCK, 10 µg/mL leupeptin, 10% glycerol, with protease inhibitors (Complete, Roche), followed by sonication for 10 s. Protein concentration was determined using Bradford reagent (Bio-Rad Laboratories). Equal amount of denatured proteins were separated by SDS-PAGE (Mini-PROTEAN TGX Gels 10%, Bio-Rad Laboratories) and transferred to nitrocellulose membranes. Membranes were blocked with 5% nonfat, dry milk in Tris-buffered saline with 0.05% Tween-20 for 1 h at 25°C, and then incubated with primary antibodies overnight at 4°C with Rabbit anti-CD36 mAb (ab133625), 1/1000, (Abcam) and Rabbit anti-β-actin(13E5) mAb #4970S, 1/1000 (Cell Signaling Technology), washed and incubated with secondary antibodies for 2 h at 25°C (Goat Anti-Rabbit IgG (H + L)-HRP Conjugate, 1/2000 (Bio-Rad Laboratories)). After washing, Amersham ECL Select Western Blotting Detection Reagent (GE Healthcare Life Sciences) was added and the light signal was detected and analyzed using a Bio-Rad Gel Doc system (Bio-Rad Laboratories).
